# Beta-cell compensation and gestational diabetes

**DOI:** 10.1016/j.jbc.2023.105405

**Published:** 2023-10-29

**Authors:** Taofeek O. Usman, Goma Chhetri, Hsuan Yeh, H. Henry Dong

**Affiliations:** Division of Endocrinology, Department of Pediatrics, University of Pittsburgh School of Medicine, Pittsburgh, Pennsylvania, USA

**Keywords:** β-cell compensation, β-cell mass, insulin resistance, gestational diabetes

## Abstract

Gestational diabetes mellitus (GDM) is characterized by glucose intolerance in pregnant women without a previous diagnosis of diabetes. While the etiology of GDM remains elusive, the close association of GDM with increased maternal adiposity and advanced gestational age implicates insulin resistance as a culpable factor for the pathogenesis of GDM. Pregnancy is accompanied by the physiological induction of insulin resistance in the mother secondary to maternal weight gain. This effect serves to spare blood glucose for the fetus. To overcome insulin resistance, maternal β-cells are conditioned to release more insulin into the blood. Such an adaptive response, termed β-cell compensation, is essential for maintaining normal maternal metabolism. β-cell compensation culminates in the expansion of β-cell mass and augmentation of β-cell function, accounting for increased insulin synthesis and secretion. As a result, a vast majority of mothers are protected from developing GDM during pregnancy. In at-risk pregnant women, β-cells fail to compensate for maternal insulin resistance, contributing to insulin insufficiency and GDM. However, gestational β-cell compensation ensues in early pregnancy, prior to the establishment of insulin resistance in late pregnancy. How β-cells compensate for pregnancy and what causes β-cell failure in GDM are subjects of investigation. In this mini-review, we will provide clinical and preclinical evidence that β-cell compensation is pivotal for overriding maternal insulin resistance to protect against GDM. We will highlight key molecules whose functions are critical for integrating gestational hormones to β-cell compensation for pregnancy. We will provide mechanistic insights into β-cell decompensation in the etiology of GDM.

Gestational diabetes mellitus (GDM) is manifested by glucose intolerance in pregnant women without a previous diagnosis of diabetes (1). GDM affects up to 10% of all pregnancies in the United States, and its prevalence is markedly higher among women with maternal obesity or advanced age at the time of gestation (2). During the COVID-19 pandemic, the incidence rate of GDM was further increased by about 13% annually (3, 4, 5). Being one of the most common obstetrical complications (2), GDM is the leading cause for preeclampsia, preterm birth, macrosomia, and cesarean delivery (6). Although GDM is transient, as a vast majority of cases with GDM recover after parturition, GDM imposes a long-lasting adverse impact on the health of both mothers and infants (1, 7, 8). Women with the first diagnosis of GDM are predisposed to developing recurrent GDM in subsequent pregnancies (9). Furthermore, women who experience GDM are seven times more likely to develop type 2 diabetes within 5 years postpartum (10, 11, 12, 13, 14, 15). Babies born to mothers with GDM are at heightened risk of developing childhood obesity or type 2 diabetes later in life (16, 17, 18). Nonetheless, the underlying etiology of GDM is poorly understood.

β-cell compensation is an adaptive mechanism by which β-cells release more insulin in response to insulin resistance or metabolic stress (19, 20). β-cell compensation is orchestrated by β-cell mass expansion, increased insulin synthesis and secretion, enhanced glucose sensing, and augmented antioxidative function in β-cells (Fig. 1). These four distinct mechanisms act alone or in concert to overcome insulin resistance for maintaining normoglycemia in insulin-resistant subjects with obesity (21, 22). Likewise, β-cell compensation ensues in response to pregnancy (23, 24, 25). Pregnancy is accompanied by enhanced insulin sensitivity in the mother in the first trimester. Such transient elevation of maternal insulin sensitivity serves to store nutrients in early pregnancy to meet maternal and fetal-placental demand for fuel metabolism in late gestation (26, 27). However, maternal insulin sensitivity declines progressively in the second trimester, resulting in overt insulin resistance in the third trimester of pregnancy (1, 26, 28, 29, 30). This effect is due to a combination of maternal weight gain and placental growth hormone, a genetic variant of growth hormone that is secreted from the placenta into the maternal circulation with an insulin-desensitizing effect on peripheral tissues in the mother (31, 32). Placental production of placental growth hormone is markedly upregulated in the third trimester, coinciding with the induction of maternal insulin resistance in late pregnancy (Fig. 2) (2, 31, 33). It is thought that such physiological induction of peripheral insulin resistance in the mother serves as a protective mechanism for sparing glucose and nutrient supplies to the developing fetus. In response to maternal insulin resistance, pancreatic islets of the mother undergo adaptive changes in β-cell mass and function, accounting for basal and postprandial increases of insulin secretion in the blood. Therefore, gestational β-cell compensation is essential for overcoming maternal insulin resistance to maintain normal glucose homeostasis during pregnancy (2, 34). Insufficient β-cell compensation for maternal insulin resistance contributes to reduced insulin secretion and impaired glucose tolerance, characteristic of GDM (2, 34).

**Figure 1:**
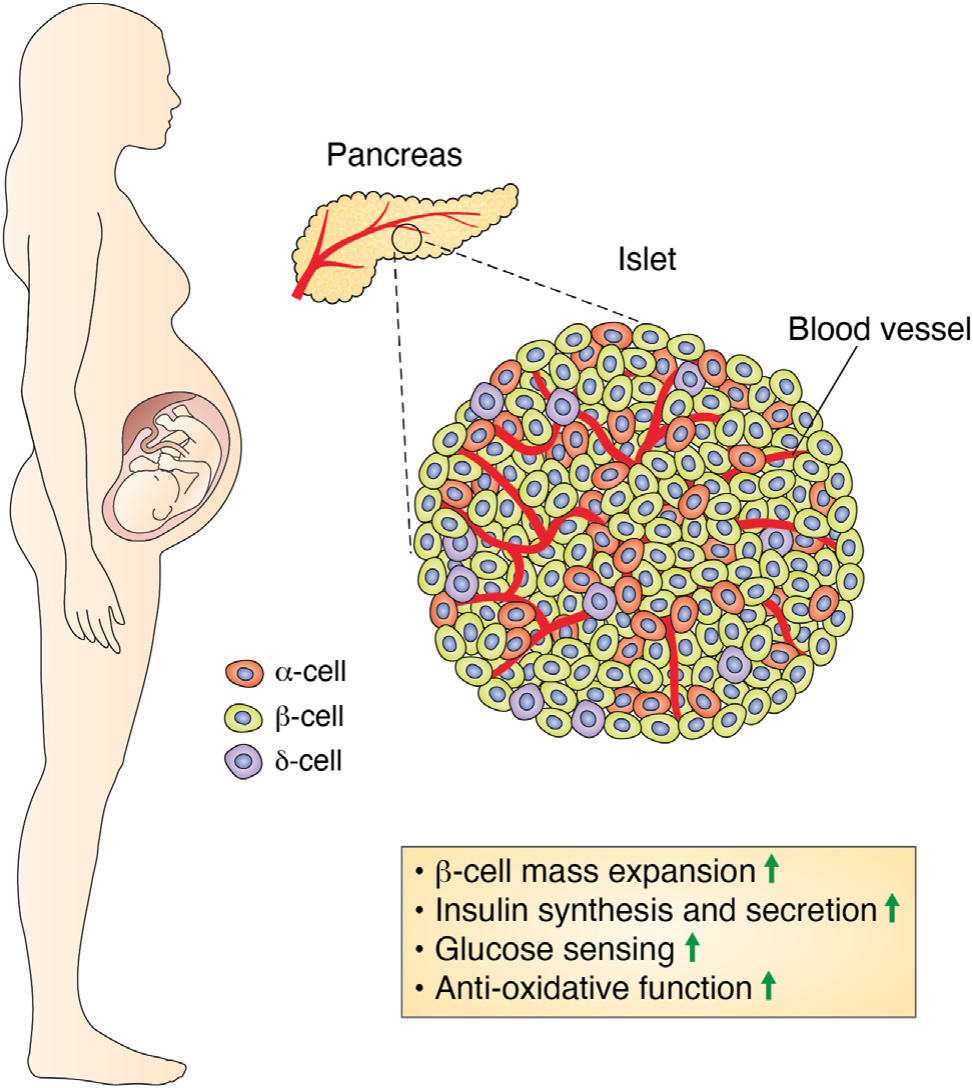
**Gestational β-cell compensation.** Pregnancy begets maternal β-cell compensation, an adaptive mechanism by which β-cells evolve to release more insulin into the blood. Gestational β-cell compensation culminates in β-cell mass expansion, increased insulin synthesis and secretion, enhanced glucose sensing, and augmented antioxidative function in maternal islets. Gestational β-cell compensation is instrumental for overcoming maternal insulin resistance to maintain normal glucose homeostasis in women during pregnancy.

**Figure 2:**
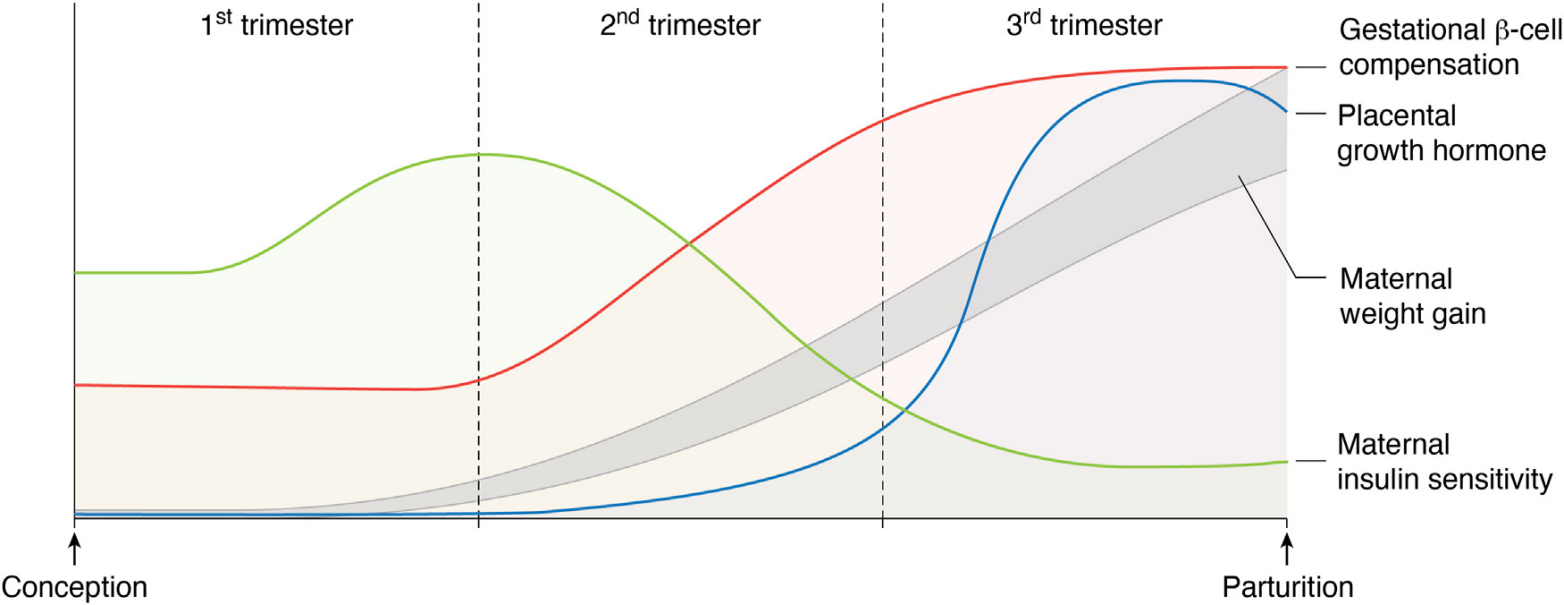
**β-cell compensation for maternal insulin resistance during pregnancy**. Pregnancy is accompanied by enhanced insulin sensitivity in the first trimester, followed by a progressive decline in maternal insulin sensitivity, resulting in overt insulin resistance in the third trimester. This effect is due to a combination of maternal weight gain and placental growth hormone, whose paracrine action contributes to insulin resistance in peripheral tissues in the mother. Gestational β-cell compensation, characterized by increased insulin secretion, initiates at the beginning of the second trimester and increases progressively to a peak level in the third trimester.

While insulin resistance begets β-cell compensation in obesity, gestational β-cell compensation ensues at the beginning of the second trimester, prior to the establishment of overt insulin resistance in the third trimester of pregnancy (Fig. 2) (1, 24, 25, 26, 28, 29, 30). This implies that β-cell compensation for pregnancy, which is different from β-cell compensation for obesity, is governed by a distinct mechanism. In this article, we will provide an in-depth review of the cellular and molecular mechanisms by which maternal islets undergo β-cell compensation for pregnancy in animal models and human subjects. We will review key molecules whose functions are instrumental for integrating gestational hormones to β-cell compensation for pregnancy. Furthermore, we will highlight the knowledge gap to inspire further clinical and preclinical investigation into the disease mechanism of GDM.

## Clinical significance of β-cell compensation for pregnancy

Clinical evidence of gestational β-cell compensation derives from longitudinal studies in pregnant women, showing that pregnancy is accompanied by a significant elevation in basal and postprandial plasma insulin levels (35, 36, 37, 38). This effect increases with the progression of gestation, indicative of an increased metabolic demand for maternal insulin during pregnancy (10). Further physiological underpinning for gestational β-cell compensation stems from the clinical observations that pregnant women with long-standing type 1 diabetes experience a relatively higher rate of hypoglycemic episodes in the first trimester or have a significantly reduced daily insulin requirement along with better glycemic control during pregnancy (39). This effect is due to endogenous insulin production in the pancreas, suggesting that pregnancy results in the recovery of residual β-cell mass and function. Of striking significance, this significant improvement in residual functional β-cell mass, as evidenced by elevated basal and postprandial insulin secretion in the blood, persists in mothers with type 1 diabetes for up to 8 weeks in the postpartum phase (39). Although the mechanism underlying the beneficial effect of pregnancy on residual β-cell function and glycemic control in type 1 diabetes remains to be elucidated (40), these clinical data lend additional support for the importance of β-cell compensation for pregnancy in women with long-standing type 1 diabetes.

Apart from physiological evidence of gestational β-cell compensation, morphological changes in islets are detectable in the pancreas of pregnant women. Van Assche *et al.* first performed a quantitative morphometric analysis of endocrine tissues on the pancreas autopsied from women with (n = 5) and without (n = 5) pregnancy (41). This assay revealed a 2-fold enlargement of endocrine area, along with a small but significant increase (∼14%) of β-cell area within the pancreas of pregnant *versus* nonpregnant women. This clinical study was subsequently advanced by Butler *et al.*, who performed insulin immunohistochemistry on pancreas tissues autopsied from women in pregnancy (n = 19), postpartum (n = 6), and nonpregnancy (n = 20), followed by morphometric analysis (42). This study reports that pregnancy is associated with about a 1.4-fold increase in islet area, without detectable changes in β-cell size or β-cell number per islet in the pancreas of pregnant women. Anti-Ki67 immunohistochemistry in combination with terminal deoxynucleotidyl transferase dUTP nick end labeling staining did not reveal significant changes in β-cell replication and apoptosis in the pancreas of pregnant women *versus* nonpregnant controls. These results suggest that clinical β-cell compensation for pregnancy is characterized by a relatively small increment (∼40%) in β-cell mass in pregnant women (42).

It is noteworthy that this study was performed on pancreas autopsies from subjects with a wide distribution of gestational length ranging from the 10th to 40th weeks of pregnancy. This may dilute the effect of pregnancy on β-cell mass, as gestational β-cell proliferation concomitant with β-cell mass expansion, takes place in late pregnancy (34, 43, 44). In addition, the assessment of maternal β-cell mass was based on β-cell area *in situ* in cross-sectional areas of the pancreas. Although not reported in this study, the pancreas weight or size tends to be larger in pregnant *versus* age-matched nonpregnant subjects. This would translate into an increase in overall maternal β-cell mass. Indeed, pregnancy is coupled with about 50% increase of pancreas weight in mice (45). Thus, the relatively small increase in maternal β-cell mass (∼1.4-fold) may be an underestimate of gestational β-cell compensation for pregnancy in humans. Instead, Butler *et al.* report a 2- to 3-fold increase in insulin-positive ductal cells and individual insulin-positive cells in the exocrine pancreas of pregnant women. These data argue for the idea that clinical β-cell compensation for pregnancy is not due to β-cell hyperplasia but is derived largely from β-cell neogenesis (42).

In support of β-cell neogenesis in gestational β-cell compensation, Dirice *et al.* visualized a 3-fold increase of insulin-positive clusters that appear to be budding from the ducts in the cadaveric pancreas from pregnant women (n = 4) (46). It is noteworthy that these duct-derived insulin-positive clusters are also glucagon-positive, characteristic of immature and progenitor endocrine cell types. This begs the question as to whether these immature dual hormonal cluster cells are responsible for the induction of gestational β-cell compensation in humans.

## β-cell mass in gestational β-cell compensation

Due to ethical concerns, it is technically impossible to probe the molecular mechanism of gestational β-cell compensation in women during pregnancy. Mechanistic insights into gestational β-cell compensation are gained mainly from studies in animal models. There is preclinical evidence that maternal islets undergo β-cell replication, resulting in a 2- to 3-fold increase of β-cell mass during pregnancy. This effect contributes to the enlargement of islets (islet hypertrophy) secondary to increased numbers of β-cells per islet (β-cell hyperplasia), accounting for enhanced glucose-stimulated insulin secretion from maternal islets. This finding is recapitulated in rats and mice during pregnancy and mice with gestational hormone-elicited pseudopregnancy (43, 47, 48, 49). It follows that β-cell mass expansion, characterized by increased β-cell replication, constitutes a major mechanism of β-cell compensation for pregnancy. While there is anecdotal evidence that new β-cells derived from ductal cells are detectable in the pancreas of pregnant mice (45), due to the lack of follow-up studies, it remains unclear whether β-cell neogenesis is an integral component of β-cell compensation for pregnancy.

## β-cell glucose sensing in gestational β-cell compensation

Glucokinase (GK) catalyzes the conversion of glucose to glucose-6-phosphate, the rate-limiting step in glycolysis (50). Glucose transporter 2 (GLUT2) functions as a low-affinity glucose transporter to facilitate postprandial glucose uptake in β-cells (51). Expressed abundantly in β-cells, GK and GLUT2 constitute the glucose-sensing mechanism that is responsible for regulating glucose-stimulated insulin secretion in the pancreas (52). GK deficiency impedes β-cell compensation for insulin resistance in dietary obese mice (53). Genetic GLUT2 mutations are associated with defective insulin secretion and neonatal diabetes in humans (54). β-cell GLUT2 is upregulated by forkhead box O1 (FoxO1), a key transcription factor that augments β-cell compensation for fat-induced insulin resistance in mice (55). These results underscore the physiological importance of glucose sensing in regulating β-cell mass and function, suggesting that perturbation of the glucose sensing mechanism in β-cells, resulting from GK or GLUT2 deficiency, is invariably associated with defective β-cell compensation. In this context, Weinhaus *et al.* show that β-cell GK activity along with GLUT2 expression in maternal islets is significantly upregulated, correlating with increased β-cell glucose oxidation and increased glucose-stimulated insulin secretion in pregnant rats (56). These effects are mediated by gestational hormones, as prolactin treatment results in a significant upregulation of GK activity and GLUT2 expression in cultured murine islets (56). These results indicate that pregnancy is coupled with enhanced β-cell glucose sensing, which contributes to gestational β-cell compensation in maternal islets.

## β-cell size in gestational β-cell compensation

β-cell mass expansion, characteristic of β-cell compensation, results from β-cell hyperplasia, β-cell hypertrophy, or a combination of both. While β-cell hyperplasia stemming from β-cell replication contributes to β-cell mass expansion, the fractional contribution of β-cell hypertrophy to the overall β-cell compensation remains a subject of debate. Rieck *et al.* report that pregnant mice have a 3-fold increase in β-cell size within islets, when compared to age-matched nonpregnant controls (48). However, this finding has not been consistently reproduced in clinical and preclinical studies. Butler *et al.* show that β-cell size within individual islets remains unchanged in the pancreas of pregnant *versus* nonpregnant women (42). Likewise, Saisho *et al.* show that despite a significant increase in β-cell mass (∼50%) in human subjects with obesity (BMI ≥ 27 kg/m^2^), as opposed to lean subjects (BMI < 25 kg/m^2^), β-cell size is similar between lean and obese subjects (57). Zhang *et al.* generated a β-cell–specific FoxO1-transgenic model with augmented β-cell compensation for obesity, demonstrating that FoxO1-transgenic mice exhibit a 3-fold induction of β-cell mass expansion in response to overnutrition. This effect is attributable to increased β-cell replication without significant alterations in β-cell size per islet in FoxO1-transgenic *versus* wildtype littermates (55). Millette *et al.* developed a mouse model with pseudopregnancy through subcutaneous administration of estrogen and placental lactogen, showing that pseudopregnant mice are associated with a significant induction in β-cell proliferation without changes in β-cell size within islets (49). Thus, it remains an open question as to whether β-cells undergo hypertrophy and what is the underlying physiology of β-cell hypertrophy.

## Oxidative stress in β-cell compensation

Clinical and preclinical studies show that pregnancy is associated with elevated nonesterified fatty acid levels. This effect, stemming from increased lipolysis secondary to reduced insulin action in adipose tissue, contributes in part to peripheral insulin resistance in the mother (31). Concomitant with the onset of maternal insulin resistance is the development of low-grade inflammation in the third trimester of pregnancy, as manifested by a marked elevation in plasma C-reactive protein and proinflammatory cytokines interleukin 1beta, interleukin 6, and tumor necrosis factor alpha in the blood (58, 59, 60, 61, 62). Women with GDM, as opposed to women without GDM, display abnormally higher serum levels of monocyte chemoattractant protein-1, a biomarker of systemic inflammation (63, 64). It follows that pregnancy, accompanied by maternal insulin resistance and low-grade inflammation along with elevated plasma nonesterified fatty acid levels, inadvertently imparts oxidative stress to β-cells in the mother. β-cells are poorly equipped with antioxidative function, due to relatively low expression of antioxidative enzymes such as superoxide dismutase, catalase, and glutathione peroxidase. As a result, β-cells are sensitive to oxidative stress. Chronic oxidative stress is thought to be an underlying cause of glucolipotoxicity for β-cell dysfunction in type 2 diabetes (65, 66, 67).

In response to metabolic stress, β-cell antioxidative function is upregulated in islets, contributing to β-cell compensation for insulin resistance in obesity (55). This is exemplified in two independent studies showing that β-cell compensation for dietary insulin resistance and obesity is regulated by the nuclear factor erythroid 2-related factor (NRF2), a basic leucine-zipper transcription factor that acts through its key targets including superoxide dismutase, catalase, and glutathione peroxidase 1 to defend cells against oxidative stress (68, 69, 70). β-cell NRF2 depletion is associated with a significant induction of β-cell apoptosis secondary to oxidative stress and reduction of β-cell proliferation, contributing to the diminution of β-cell mass and onset of prediabetes in HFD-fed β-cell NFR2-KO mice (69). Conversely, genetic and pharmacological activation of NRF2 activity suppresses β-cell apoptosis and increases β-cell proliferation in murine and human islets that are transplanted under the kidney capsule of immunodeficient mice (69). Notwithstanding the importance of β-cell oxidative stress in the adaptive changes of β-cell mass and function in obesity, it remains unknown whether maternal oxidative stress impacts β-cell compensation for maternal insulin resistance in pregnancy. To answer this fundamental question, Gurlo *et al.* generated transgenic mice with β-cell-specific production of human islet amyloid polypeptide, whose aggregation is linked to β-cell dysfunction secondary to oxidative stress in humans with type 2 diabetes (71). Transgenic human islet amyloid polypeptide overproduction results in undue oxidative stress in β-cells, rendering female mice susceptible to developing GDM (71). These data implicate excessive oxidative stress as a confounding factor for the pathogenesis of GDM.

Further physiological underpinning for the link of β-cell oxidative stress with GDM derives from a study by Prentice *et al.*, who report that the furan fatty acid metabolite 3-carboxy-4-methyl-5-propyl-2-furanpropanoic acid (CMPF) is markedly elevated in the plasma of women with GDM and patients with type 2 diabetes (72). When administered into normal mice, CMPF blunts glucose-stimulated insulin release, resulting in glucose intolerance. When added in culture medium, CMPF inhibits glucose-stimulated insulin secretion in primary human and murine islets and MIN6 cells. Mechanistically, CMPF enters β-cells *via* the organic anion transporter-3 (OAT3) to impair mitochondrial function with a concomitant induction of reactive oxygen species production and reduction of glucose-stimulated ATP accumulation in β-cells. These findings are recapitulated by Liu *et al.*, who show that administration of CMPF exacerbates the development of diabetes in dietary obese mice and genetic *ob/ob* mice, due in part to CMPF-mediated mitochondrial reactive oxygen species production and oxidative stress in islets (73). However, this deleterious effect of CMPF on β-cell function is abrogated by blocking CMPF entrance to β-cells *via* genetic OAT3 depletion or pharmacological OAT3 inhibition (72). These results underscore the pathological significance of β-cell oxidative stress in GDM and type 2 diabetes (74).

## β-cell apoptosis in GDM

Is β-cell apoptosis a causative factor for the pathogenesis of GDM? To answer this fundamental question, Butler *et al.* employed the terminal deoxynucleotidyl transferase dUTP nick end labeling immunostaining approach to visualize apoptotic β-cells in the pancreas autopsies of women with GDM *versus* normal pregnancy (42). This assay did not reveal significant differences in the number of apoptotic β-cells in the pancreas between groups. Furthermore, the overall incidence of detectable β-cell apoptosis is extremely low in the pancreas in both groups. Due to rapid turnover, apoptotic cells are efficiently cleared by the immune system *in vivo*, posing a technical challenge to visualize *in situ* apoptotic β-cells in the pancreas. To overcome this technical limitation, Akirav *et al.* developed a method for detecting β-cell death *in vivo*, using quantitative PCR assay to measure β-cell-derived demethylated preproinsulin DNA in the blood (75). They then applied this assay to measure β-cell death in pregnant women with normal pregnancy (n = 14) or GDM (n = 22), demonstrating that the detectable events of β-cell death are significantly lower in women with GDM *versus* normal pregnancy (76). Together these results argue against the idea that β-cell apoptosis is a contributor for the pathogenesis of GDM in humans.

## Mechanism of β-cell compensation in pregnancy

Pregnancy is accompanied by the production of prolactin and placental lactogen, two gestational hormones whose paracrine actions *via* prolactin receptor (PRLR) in maternal islets are instrumental for augmenting β-cell compensation for pregnancy (77). There is clinical evidence that women harboring genetic mutations in the 5′-untranslated region or coding region of the PRLR gene are at a higher risk of developing GDM (78, 79). Preclinical studies indicate that β-cell conditional PRLR depletion impairs gestational β-cell compensation, resulting in the onset of GDM in pregnant mice (80, 81). PRLR haploinsufficiency predisposes female mice to GDM, due to insufficient β-cell compensation during pregnancy (82, 83). PRLR expression in β-cells is markedly upregulated in response to gestation, correlating with the gestational surge of prolactin and placental lactogen production in pregnant mice (47, 48). Hormonal activation of PRLR signaling stimulates β-cell proliferation in islets, and this effect contributes to increased β-cell mass and enhanced glucose-stimulated insulin secretion in mice (84, 85). These data illustrate an obligatory role of PRLR in augmenting gestational β-cell compensation to meet increased metabolic demand for insulin during pregnancy. Nonetheless, the molecular basis that integrates PRLR signaling to β-cell mass expansion in pregnancy remains elusive.

**Figure 3:**
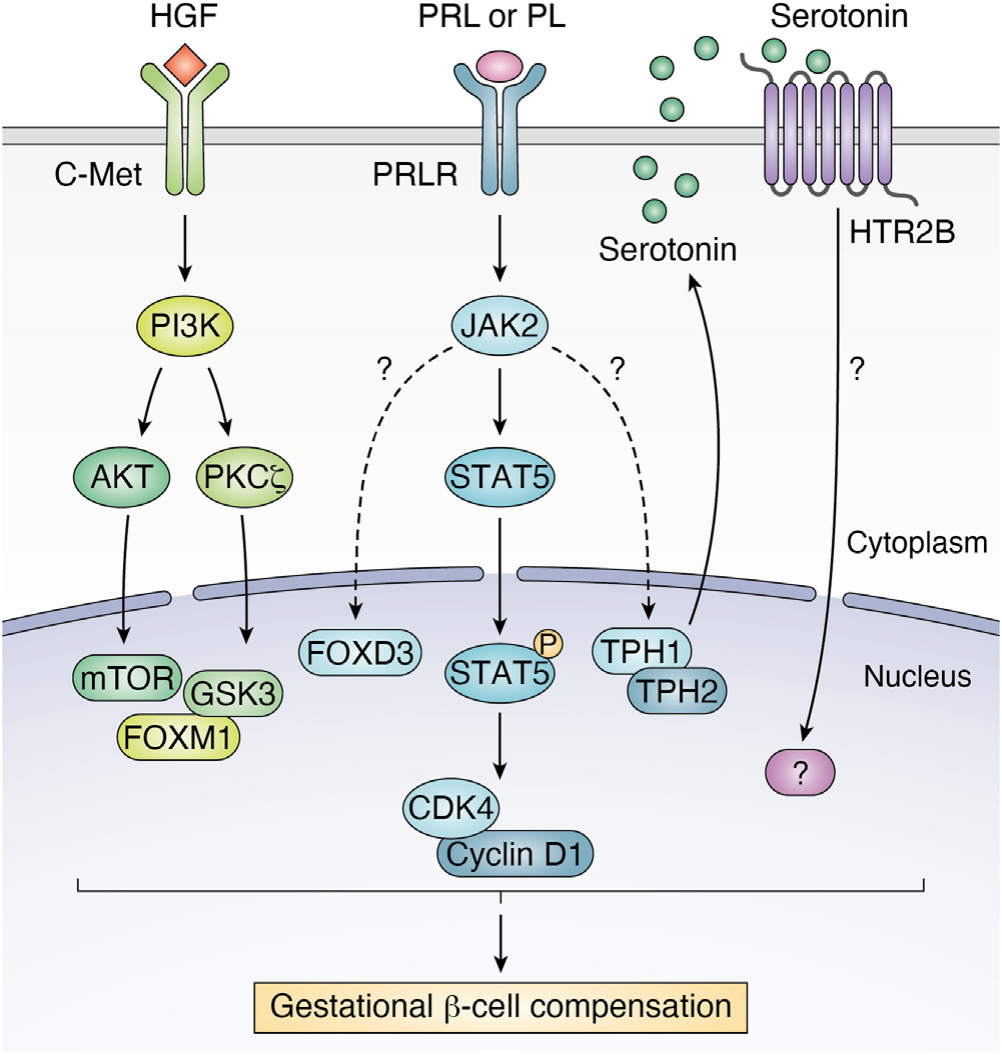
**Mechanism of β-cell compensation for pregnancy.** β-cells compensate for maternal insulin resistance in multiple mechanisms. First, PRL- and PL-mediated activation of PRLR signaling stimulates STAT5 phosphorylation by JAK2. This effect promotes STAT5 translocation from the cytoplasm to the nucleus and augments STAT5 activity in stimulating β-cell proliferation. Second, HGF-mediated activation of C-Met signaling functions *via* PI3K to enhance AKT and PKCζ activities. This effect promotes β-cell production of mTOR, GSK3, and FOXM1, key proteins in stimulating β-cell proliferation. Third, PRL- and PL-mediated activation of PRLR signaling is associated with increased β-cell production of serotonin, whose autocrine action *via* HTR2B contributes to β-cell mass expansion during pregnancy. The *question mark* denotes the signaling pathway to be delineated. AKT, serine/threonine protein kinase; CDK4, cyclin-dependent kinase 4; C-Met, mesenchymal-epithelial transition factor; FoxM1, forkhead box M1; FoxD3, forkhead box D3; GSK3, glycogen synthase kinase 3; HGF, hepatocyte growth factor; JAK2, Janus kinase 2, mTOR, mammalian target of rapamycin; PI3K, phosphoinositide 3-kinase; PKCζ, protein kinase C ζ; PL, placenta lactogen; PRLR, prolactin receptor; PRL, prolactin; STAT5, signal transducer and activator of transcription five; TPH-1, tryptophan hydrolase-1; TPH-2, tryptophan hydrolase-2.

PRLR is a type-1 cytokine receptor that signals through Janus kinase 2 to stimulate the phosphorylation and activation of signal transducer and activator of transcription 5 (STAT5), a key transcription factor that is shown to mediate β-cell proliferation (86, 87) (Fig. 3). This has led to the idea that STAT5 is the downstream effector of PRLR signaling to mediate the proliferative effect of gestational hormones on β-cell mass expansion in pregnancy. However, β-cell conditional STAT5 depletion has no discernible impacts on β-cell growth and differentiation (88). As a result, pregnant mice with β-cell STAT5 depletion have normal maternal metabolism, defying the expectation that β-cell STAT5 is responsible for gestational β-cell compensation. Alternatively, Kim *et al.* report that serotonin acts downstream of PRLR signaling to regulate β-cell compensation for pregnancy (43). In response to gestation, β-cell production of serotonin along with its receptor Htr2B is markedly increased, and this effect stimulates β-cell proliferation, contributing to β-cell mass expansion in pregnant mice. Blocking serotonin-Htr2B signaling inhibits β-cell mass expansion, resulting in glucose intolerance in pregnant mice. These results suggest that serotonin signaling *via* Htr2B plays a role in modulating β-cell adaptation to pregnancy, although the underlying mechanism by which serotonin acts to stimulate β-cell proliferation remains to be elucidated (24). It is noteworthy that serotonin synthesis is catalyzed by tryptophan hydrolase-1 (TPH-1), a predominant isoform in β-cells. However, female mice with genetic depletion of TPH-1 or Htr2B have similar degrees of β-cell mass expansion and glucose tolerance as wildtype littermates during pregnancy (89). Adding to the controversy over the role of serotonin signaling in β-cell compensation for pregnancy is the clinical evidence that neither TPH-1 nor Htr2B polymorphism is associated with the risk of GDM in humans (90). Thus, it remains an open question as to how activation of PRLR signaling translates into the proliferative effect on β-cells, contributing to gestational β-cell compensation for pregnancy.

## Genetic factors in β-cell decompensation and GDM

β-cell decompensation, characterized by the disabilities of β-cells to compensate for maternal insulin resistance, presages the onset of GDM. While the underlying etiology remains elusive, the fact that GDM develops in some (∼10%), but not in all pregnant women in spite of prevailing maternal insulin resistance, forebodes an intricate interplay between genetic factors and nutritional cues in β-cell decompensation in GDM. Although increased maternal adiposity and advanced gestational age compound the development of GDM, genetic factors whose altered expression or function in β-cells are linked to GDM are incompletely characterized. Indeed, apart from PRLR and serotonin, only a few genetic factors including hepatocyte growth factor (HGF), Menin, forkhead box M1 (FoxM1), and forkhead box D3 (FoxD3) have been linked to the etiology of GDM.

Similar to the gestational surge of prolactin in the blood, plasma HGF levels are markedly upregulated during pregnancy. To understand the underlying physiology, Demirci *et al.* generated a mouse model with pancreas-conditional knockout of mesenchymal-epithelial transition factor (C-Met), the cognate receptor of HGF (91). C-Met deficiency is associated with decreased β-cell replication and increased β-cell apoptosis in maternal islets. This effect results in β-cell decompensation, as reflected in the diminution of β-cell mass and reduction of glucose-stimulated insulin secretion in C-Met deficient islets. As a result, female pancreas-conditional C-Met knockout mice are predisposed to developing GDM during pregnancy. In pregnant wildtype mice, C-Met is upregulated in islets, correlating with gestational β-cell mass expansion (91). These results unveil the physiological importance of HGF-C-Met signaling in governing gestational β-cell compensation to protect against GDM. Nonetheless, the clinical significance of this finding remains unknown, as altered serum HGF levels are not associated with an increased risk of GDM in women (92).

Menin, encoded by the MEN1 gene, is a scaffold protein that functions as a tumor suppressor (93, 94). Karnik *et al.* report that transgenic Menin expression in maternal islets results in a marked upregulation of p18 and p27, two key inhibitors of mitotic cell cycle progression (44). This effect translates into an inhibitory effect on gestational β-cell compensation, resulting in the induction of fasting hyperglycemia and glucose intolerance in pregnant Menin-deficient mice. These results suggest that Menin is a negative regulator of gestational β-cell compensation. Consistent with this idea, Karnik *et al.* show that β-cell Menin expression is effectively inhibited by prolactin in normal pregnancy (44). This effect depends on PRLR-mediated induction of STAT5 activity, which promotes β-cell expression of B-cell lymphoma 6, a transcriptional inhibitor of Menin. However, this interpretation is contradicted by the observation that β-cell STAT5 depletion does not affect gestational β-cell compensation in pregnant mice (88). Increased Menin expression in islets is associated with insulinoma (93, 94). Thus, further investigation is warranted to define the mechanism by which β-cell Menin production is inhibited in maternal islets for better understanding of its role in regulating β-cell compensation for pregnancy.

FoxM1 is a transcription factor that is ubiquitously expressed in mammals. Zhang *et al.* show that β-cell FoxM1 expression is markedly upregulated in response to gestation (95). FoxM1 acts downstream of PRLR signaling to stimulate β-cell proliferation in cultured islets (95). Genetic FoxM1 depletion in the pancreas impedes β-cell proliferation during pregnancy, resulting in severe glucose intolerance in mice at gestational day 15.5 (95). Likewise, β-cell FoxM1 production is upregulated in response to obesity. This effect contributes to β-cell mass expansion in dietary obese mice (96). These results suggest that FoxM1-mediated β-cell compensation constitutes a critical mechanism that is shared by obesity and pregnancy.

FoxD3 is another transcription factor whose function is linked to gestational β-cell compensation. Plank *et al.* show that female mice with pancreas-specific FoxD3 depletion exhibit glucose intolerance during pregnancy (97). This effect is not secondary to defective glucose-stimulated insulin secretion in maternal islets, but due to the inability of maternal islets to undergo β-cell proliferation in response to gestation. FoxM1 is significantly reduced in FoxD3-deficient islets, coinciding with decreased β-cell mass in pregnant FoxD3-knockout mice. These data suggest that FoxD3 acts upstream of FoxM1 to regulate β-cell adaptation to pregnancy. However, this interpretation is at odds with the evidence that islet FoxD3 expression is markedly downregulated in pregnant *versus* virgin mice (97). Further studies are needed to characterize the role of FoxD3 in gestational β-cell compensation and determine its contribution to GDM.

## α-cell adaptation to pregnancy

There is emerging evidence that pregnancy is accompanied by α-cell mass expansion (98, 99, 100). This effect results from increased α-cell replication but not from transdifferentiation of exocrine cell types (100). However, despite a 2-fold increment of α-cell mass at gestational day 18.5, plasma glucagon levels remain unchanged in pregnant mice (98, 99, 100). To understand the underlying physiology of α-cell adaptation to pregnancy, Qiao *et al.* generated a mouse model with pharmacological ablation of α-cells (>95% α-cells depleted) in the pancreas (98). Virgin α-cell null mice exhibit better glycemic control in response to glucose challenge, but during pregnancy, α-cell null mice develop severe glucose intolerance due to significantly reduced glucose-stimulated insulin secretion. Notably, α-cell null and wildtype dams have similar β-cell mass at gestational day 18.5, indicating that islets devoid of α-cells are able to undergo normal β-cell mass expansion during pregnancy (98). These results reveal a vital role of α-cells in maternal metabolism, suggesting that α-cell depletion somewhat impairs β-cell function in maternal islets, contributing to insulin insufficiency and glucose intolerance in α-cell null dams. While α-cells make up 15 to 20% of total cells in rodent islets (30–40% of total cells in human islets), glucagon secreted from α-cells is known to act as a counterregulatory hormone at β-cells to stimulate insulin secretion (101). The observation that α-cell depletion impairs β-cell function independently of β-cell mass reinforces the idea that intraislet paracrine crosstalk between α-cells and β-cells are critical for maintaining the functional integrity of islets for effective metabolic adaptation to pregnancy. Consistent with this idea is the observation that pregnancy is concomitant with both α-cell and β-cell mass expansion to the same extent, so that the ratio of α-cells and β-cells in islets is maintained during pregnancy (98, 99, 100).

Nonetheless, this idea is contrasted by data from two independent studies showing that neonatal mice with near complete α-cell depletion grow normally with normal postnatal β-cell growth and maturation, accompanied by normal glucose-stimulated insulin secretion and normal glucose homeostasis in adulthood (102, 103). Although there is clinical evidence that plasma glucagon levels are significantly elevated in the second trimester, followed by a decline to a normal range in the third trimester in pregnant women (104), the underlying physiology of such transient elevation in plasma glucagon levels is unknown. There are no reports in the literature about α-cell adaptation to pregnancy in humans. Instead, there is preclinical evidence that plasma glucagon levels are not correlated with α-cell mass (103). Further investigation is needed to understand the physiological significance of α-cell mass expansion in metabolic adaptation to pregnancy.

## β-cell mass involution postpartum

Maternal β-cell mass, which is increased by 2- to 3-fold during pregnancy, contracts after parturition. This process, termed β-cell mass involution, stems from increased β-cell apoptosis and decreased β-cell proliferation, although there is evidence that β-cell size is also reduced in maternal islets postpartum (105). In support of this notion, Scaglia *et al.* show that pregnancy-induced β-cell mass reverses to nonpregnant levels in rats 10 days postpartum (105). Likewise, Takahashi *et al.* reveal that gestation-elicited β-cell mass decreases to prepregnancy levels in mice in 7 days postpartum (106). This effect is due to the reduction of β-cell size in maternal islets, which deviates from the prevailing idea that β-cell apoptosis is the major mechanism for postpartum β-cell mass involution (105). However, the underlying mechanism of postpartum β-cell size reduction is not disclosed (106). Furthermore, a slight reduction in β-cell size (∼30%) cannot account for the 2- to 3-fold reduction in β-cell mass in maternal islets in mice postpartum (34, 106). Therefore, it remains an open question as to how maternal β-cell mass, which is expanded by 2- to 3-fold at the end of pregnancy, undergoes rapid involution to prepregnancy levels in 7 to 10 days postpartum in rodents.

Intriguingly, Takahashi *et al.* show that maternal β-cell mass, following a transient reduction to nonpregnant levels at postnatal day 7, is increased again by about 2-fold along with enhanced glucose-stimulated insulin secretion in postpartum mice at postnatal day 21 (106). This effect is correlated with elevated prolactin levels in the blood and increased serotonin production in maternal islets in lactating dams, suggesting that lactation-induced production of prolactin and serotonin contributes to maternal β-cell mass expansion postpartum. This lactation-induced β-cell mass expansion is completely abolished in nonlactating dams (106). These results illustrate an unexpected phase of β-cell compensation in maternal islets in response to increased metabolic demand for insulin during lactation.

## Conclusion and perspective

Gestational β-cell compensation is an adaptive mechanism by which β-cells evolve to upregulate insulin secretion for overcoming maternal insulin resistance to protect against GDM. Although such a vital mechanism is evolutionarily conserved, gestational β-cell compensation differs in fundamental ways between rodents and humans. First, rodents exhibit a robust gestational β-cell compensation, accompanied by 2- to 3-fold expansion of β-cell mass at the end of pregnancy. In contrast, a relatively small increment in maternal β-cell mass (∼1.4-fold) is observed in humans in late pregnancy (42). However, this clinical observation is confounded by the lack of consideration of pancreas weight or size that is predicted to increase in late pregnancy, and the inclusion of pregnant women in early pregnancy, during which β-cell compensation is maintained at a baseline level, as opposed to late pregnancy (42). These caveats likely contribute to the underestimation of clinical β-cell compensation for pregnancy. Second, maternal β-cell mass expansion is accomplished primarily by increased β-cell replication in pregnant rodents. This is different from humans, in which β-cell neogenesis appears to account for the increment in maternal β-cell mass expansion. Third, there is anecdotal evidence that β-cell size is increased in pregnant mice (48). However, this finding has not been consistently reproduced in preclinical studies (49, 55). Furthermore, such quantitative changes in β-cell size are not detectable in pancreas autopsies of pregnant *versus* nonpregnant women (42, 57). Further investigation is needed to clarify whether β-cells undergo hypertrophy in response to gestation, contributing to β-cell compensation for pregnancy.

Although β-cell PRLR acts to mediate the proliferative effects of prolactin and placental lactogen on β-cells, the molecular events downstream of PRLR signaling in β-cells are incompletely characterized (Fig. 3). While STAT5 is characterized as a key factor for linking PRLR signaling to β-cell replication *in vitro* and *in vivo* (86, 87), β-cell STAT5 depletion has little impact on β-cell compensation for pregnancy (88). Likewise, serotonin is shown to act downstream of β-cell PRLR signaling to stimulate β-cell proliferation (43). However, genetic depletion of serotonin synthetic enzyme TPH1 or serotonin receptor Htr2b has not consistently resulted in the development of GDM in mice (43, 89). Further research is warranted to uncover alternative routes or factors that are responsible for integrating PRLR signaling with gestational β-cell mass expansion (Fig. 3).

Equally important is β-cell mass involution, the process that governs the progressive reduction of maternal β-cell mass to a normal range after parturition. The underlying mechanism is poorly understood. Although there is a consensus that β-cell proliferation in maternal islets is completely inhibited after birth, there are controversies over whether β-cell apoptosis *versus* β-cell size reduction is a major contributing factor for postpartum β-cell mass involution (105, 106). Clearly, studies are needed to resolve these controversies for better understanding of the mechanism underlying β-cell mass involution postpartum.

## Conflict of interest

The authors have no conflicts of interest with the content of this article.

## References

[1] Buchanan T.A., Xiang A.H. (2005). Gestational diabetes mellitus. J. Clin. Invest..

[2] Buchanan T.A., Xiang A.H., Page K.A. (2012). Gestational diabetes mellitus: risks and management during and after pregnancy. Nat. Rev. Endocrinol..

[3] Gregory E.C., Ely D.M. (2022). Trends and characteristics in gestational diabetes: United States, 2016-2020. Natl. Vital Stat. Rep..

[4] Zanardo V., Tortora D., Sandri A., Severino L., Mesirca P., Straface G. (2022). COVID-19 pandemic: impact on gestational diabetes mellitus prevalence. Diabetes Res. Clin. Pract..

[5] Chelu S., Bernad E., Craina M., Neamtu R., Mocanu A.G., Vernic C. (2022). Prevalence of gestational diabetes in preCOVID-19 and COVID-19 years and its impact on pregnancy: a 5-year retrospective study. Diagnostics (Basel).

[6] Wang X., Zhang X., Zhou M., Juan J., Wang X. (2021). Association of gestational diabetes mellitus with adverse pregnancy outcomes and its interaction with maternal age in Chinese urban women. J. Diabetes Res..

[7] Soares de Souza E.D.S., Saunders C., do Carmo C.N., de Aquino Lacerda E.M., Zajdenverg L., de Castro M.B.T. (2022). Gestational weight gain and adverse maternal and perinatal outcomes among women with gestational diabetes mellitus according to International Association of Diabetes and Pregnancy Study Group (IADPSG) criteria: a cross sectional study. Clin. Nutr. ESPEN.

[8] Ghosh S., Ghosh K. (2013). Maternal and neonatal outcomes in gestational diabetes mellitus. J. Indian Med. Assoc..

[9] Egan A.M., Enninga E.A.L., Alrahmani L., Weaver A.L., Sarras M.P., Ruano R. (2021). Recurrent gestational diabetes mellitus: a narrative review and single-center experience. J. Clin. Med..

[10] Xiang A.H., Takayanagi M., Black M.H., Trigo E., Lawrence J.M., Watanabe R.M. (2013). Longitudinal changes in insulin sensitivity and beta cell function between women with and without a history of gestational diabetes mellitus. Diabetologia.

[11] Lowe W.L., Scholtens D.M., Sandler V., Hayes M.G. (2016). Genetics of gestational diabetes mellitus and maternal metabolism. Curr. Diab. Rep..

[12] Radha V., Kanthimathi S., Anjana R.M., Mohan V. (2016). Genetics of gestational diabetes mellitus. J. Pak Med. Assoc..

[13] Robitaille J., Grant A.M. (2008). The genetics of gestational diabetes mellitus: evidence for relationship with type 2 diabetes mellitus. Genet. Med..

[14] Watanabe R.M., Black M.H., Xiang A.H., Allayee H., Lawrence J.M., Buchanan T.A. (2007). Genetics of gestational diabetes mellitus and type 2 diabetes. Diabetes Care.

[15] Bider-Canfield Z., Martinez M.P., Wang X., Yu W., Bautista M.P., Brookey J. (2017). Maternal obesity, gestational diabetes, breastfeeding and childhood overweight at age 2 years. Pediatr. Obes..

[16] Page K.A., Luo S., Wang X., Chow T., Alves J., Buchanan T.A. (2019). Children exposed to maternal obesity or gestational diabetes mellitus during early fetal development have hypothalamic alterations that predict future weight gain. Diabetes Care.

[17] Xiang A.H., Wang X., Martinez M.P., Getahun D., Page K.A., Buchanan T.A. (2018). Maternal gestational diabetes mellitus, type 1 diabetes, and type 2 diabetes during pregnancy and risk of ADHD in offspring. Diabetes Care.

[18] Crume T.L., Ogden L., Daniels S., Hamman R.F., Norris J.M., Dabelea D. (2011). The impact of in utero exposure to diabetes on childhood body mass index growth trajectories: the EPOCH study. J. Pediatr..

[19] Prentki M., Nolan C.J. (2006). Islet beta cell failure in type 2 diabetes. J. Clin. Invest..

[20] Sachdeva M.M., Stoffers D.A. (2009). Minireview: meeting the demand for insulin: molecular mechanisms of adaptive postnatal beta-cell mass expansion. Mol. Endocrinol..

[21] Rhodes C.J. (2005). Type 2 diabetes-a matter of beta-cell life and death?. Science.

[22] Weir G.C., Bonner-Weir S. (2004). Five stages of evolving beta-cell dysfunction during progression to diabetes. Diabetes.

[23] Sorenson R.L., Brelje T.C. (1997). Adaptation of islets of Langerhans to pregnancy: beta-cell growth, enhanced insulin secretion and the role of lactogenic hormones. Horm. Metab. Res..

[24] Baeyens L., Hindi S., Sorenson R.L., German M.S. (2016). beta-Cell adaptation in pregnancy. Diabetes Obes. Metab..

[25] Ernst S., Demirci C., Valle S., Velazquez-Garcia S., Garcia-Ocana A. (2011). Mechanisms in the adaptation of maternal beta-cells during pregnancy. Diabetes Manag. (Lond).

[26] Ryan E.A. (2003). Hormones and insulin resistance during pregnancy. Lancet.

[27] Lain K.Y., Catalano P.M. (2007). Metabolic changes in pregnancy. Clin. Obstet. Gynecol..

[28] Sonagra A.D., Biradar S.M., K D., Murthy D.S.J. (2014). Normal pregnancy- a state of insulin resistance. J. Clin. Diagn. Res..

[29] Xiang A.H., Peters R.K., Trigo E., Kjos S.L., Lee W.P., Buchanan T.A. (1999). Multiple metabolic defects during late pregnancy in women at high risk for type 2 diabetes. Diabetes.

[30] Catalano P.M., Huston L., Amini S.B., Kalhan S.C. (1999). Longitudinal changes in glucose metabolism during pregnancy in obese women with normal glucose tolerance and gestational diabetes mellitus. Am. J. Obstet. Gynecol..

[31] Barbour L.A., McCurdy C.E., Hernandez T.L., Kirwan J.P., Catalano P.M., Friedman J.E. (2007). Cellular mechanisms for insulin resistance in normal pregnancy and gestational diabetes. Diabetes Care.

[32] Barbour L.A., Shao J., Qiao L., Pulawa L.K., Jensen D.R., Bartke A. (2002). Human placental growth hormone causes severe insulin resistance in transgenic mice. Am. J. Obstet. Gynecol..

[33] McIntyre H.D., Serek R., Crane D.I., Veveris-Lowe T., Parry A., Johnson S. (2000). Placental growth hormone (GH), GH-binding protein, and insulin-like growth factor axis in normal, growth-retarded, and diabetic pregnancies: correlations with fetal growth. J. Clin. Endocrinol. Metab..

[34] Rieck S., Kaestner K.H. (2010). Expansion of beta-cell mass in response to pregnancy. Trends Endocrinol. Metab..

[35] Spellacy W.N., Goetz F.C., Greenberg B.Z., Ells J. (1965). Plasma insulin in normal "early" pregnancy. Obstet. Gynecol..

[36] Spellacy W.N., Goetz F.C. (1963). Plasma insulin in normal late pregnancy. N. Engl. J. Med..

[37] Powe C.E., Huston Presley L.P., Locascio J.J., Catalano P.M. (2019). Augmented insulin secretory response in early pregnancy. Diabetologia.

[38] Catalano P.M., Drago N.M., Amini S.B. (1998). Longitudinal changes in pancreatic beta-cell function and metabolic clearance rate of insulin in pregnant women with normal and abnormal glucose tolerance. Diabetes Care.

[39] Espes D., Magnusson L., Caballero-Corbalan J., Schwarcz E., Casas R., Carlsson P.O. (2022). Pregnancy induces pancreatic insulin secretion in women with long-standing type 1 diabetes. BMJ Open Diabetes Res. Care.

[40] Nalla A., Ringholm L., Sorensen S.N., Damm P., Mathiesen E.R., Nielsen J.H. (2020). Possible mechanisms involved in improved beta cell function in pregnant women with type 1 diabetes. Heliyon.

[41] Van Assche F.A., Aerts L., De Prins F. (1978). A morphological study of the endocrine pancreas in human pregnancy. Br. J. Obstet. Gynaecol..

[42] Butler A.E., Cao-Minh L., Galasso R., Rizza R.A., Corradin A., Cobelli C. (2010). Adaptive changes in pancreatic beta cell fractional area and beta cell turnover in human pregnancy. Diabetologia.

[43] Kim H., Toyofuku Y., Lynn F.C., Chak E., Uchida T., Mizukami H. (2010). Serotonin regulates pancreatic beta cell mass during pregnancy. Nat. Med..

[44] Karnik S.K., Chen H., McLean G.W., Heit J.J., Gu X., Zhang A.Y. (2007). Menin controls growth of pancreatic beta-cells in pregnant mice and promotes gestational diabetes mellitus. Science.

[45] Abouna S., Old R.W., Pelengaris S., Epstein D., Ifandi V., Sweeney I. (2010). Non-beta-cell progenitors of beta-cells in pregnant mice. Organogenesis.

[46] Dirice E., De Jesus D.F., Kahraman S., Basile G., Ng R.W., El Ouaamari A. (2019). Human duct cells contribute to beta cell compensation in insulin resistance. JCI Insight.

[47] Parsons J.A., Brelje T.C., Sorenson R.L. (1992). Adaptation of islets of Langerhans to pregnancy: increased islet cell proliferation and insulin secretion correlates with the onset of placental lactogen secretion. Endocrinology.

[48] Rieck S., White P., Schug J., Fox A.J., Smirnova O., Gao N. (2009). The transcriptional response of the islet to pregnancy in mice. Mol. Endocrinol..

[49] Millette K., Rodriguez K., Sheng X., Finley S.D., Georgia S. (2022). Exogenous lactogenic signaling stimulates beta cell replication *in vivo* and *in vitro*. Biomolecules.

[50] Ashcroft F.M., Lloyd M., Haythorne E.A. (2023). Glucokinase activity in diabetes: too much of a good thing?. Trends Endocrinol. Metab..

[51] Thorens B. (2015). GLUT2, glucose sensing and glucose homeostasis. Diabetologia.

[52] Weir G.C., Laybutt D.R., Kaneto H., Bonner-Weir S., Sharma A. (2001). Beta-cell adaptation and decompensation during the progression of diabetes. Diabetes.

[53] Terauchi Y., Takamoto I., Kubota N., Matsui J., Suzuki R., Komeda K. (2007). Glucokinase and IRS-2 are required for compensatory beta cell hyperplasia in response to high-fat diet-induced insulin resistance. J. Clin. Invest..

[54] Sansbury F.H., Flanagan S.E., Houghton J.A., Shuixian Shen F.L., Al-Senani A.M., Habeb A.M. (2012). SLC2A2 mutations can cause neonatal diabetes, suggesting GLUT2 may have a role in human insulin secretion. Diabetologia.

[55] Zhang T., Kim D.H., Xiao X., Lee S., Gong Z., Muzumdar R. (2016). FoxO1 plays an important role in regulating beta-cell compensation for insulin resistance in male mice. Endocrinology.

[56] Weinhaus A.J., Stout L.E., Sorenson R.L. (1996). Glucokinase, hexokinase, glucose transporter 2, and glucose metabolism in islets during pregnancy and prolactin-treated islets *in vitro*: mechanisms for long term up-regulation of islets. Endocrinology.

[57] Saisho Y., Butler A.E., Manesso E., Elashoff D., Rizza R.A., Butler P.C. (2013). beta-cell mass and turnover in humans: effects of obesity and aging. Diabetes Care.

[58] Parisi F., Milazzo R., Savasi V.M., Cetin I. (2021). Maternal low-grade chronic inflammation and intrauterine programming of health and disease. Int. J. Mol. Sci..

[59] de Castro J., Sevillano J., Marciniak J., Rodriguez R., Gonzalez-Martin C., Viana M. (2011). Implication of low level inflammation in the insulin resistance of adipose tissue at late pregnancy. Endocrinology.

[60] Xuan Nguyen K., Bui Minh T., Dinh H.T., Viet Tran T., Dinh Le T., Phi Thi Nguyen N. (2023). Low-grade inflammation in gestational diabetes mellitus and its correlation with maternal insulin resistance and fetal growth indices. Int. J. Gen. Med..

[61] Fink N.R., Chawes B., Bonnelykke K., Thorsen J., Stokholm J., Rasmussen M.A. (2019). Levels of systemic low-grade inflammation in pregnant mothers and their offspring are correlated. Sci. Rep..

[62] Bo S., Signorile A., Menato G., Gambino R., Bardelli C., Gallo M.L. (2005). C-reactive protein and tumor necrosis factor-alpha in gestational hyperglycemia. J. Endocrinol. Invest..

[63] Klein K., Satler M., Elhenicky M., Brix J., Krzyzanowska K., Schernthaner G. (2008). Circulating levels of MCP-1 are increased in women with gestational diabetes. Prenat Diagn..

[64] Liu H., Liu A., Kaminga A.C., McDonald J., Wen S.W., Pan X. (2022). Chemokines in gestational diabetes mellitus. Front. Immunol..

[65] Robertson R.P. (2004). Chronic oxidative stress as a central mechanism for glucose toxicity in pancreatic islet beta cells in diabetes. J. Biol. Chem..

[66] Martens G.A., Cai Y., Hinke S., Stange G., Van de Casteele M., Pipeleers D. (2005). Glucose suppresses superoxide generation in metabolically responsive pancreatic beta cells. J. Biol. Chem..

[67] Poitout V., Robertson R.P. (2008). Glucolipotoxicity: fuel excess and beta-cell dysfunction. Endocr. Rev..

[68] Yagishita Y., Fukutomi T., Sugawara A., Kawamura H., Takahashi T., Pi J. (2014). Nrf2 protects pancreatic beta-cells from oxidative and nitrosative stress in diabetic model mice. Diabetes.

[69] Baumel-Alterzon S., Katz L.S., Brill G., Jean-Pierre C., Li Y., Tse I. (2022). Nrf2 regulates beta-cell mass by suppressing beta-cell death and promoting beta-cell proliferation. Diabetes.

[70] Baumel-Alterzon S., Katz L.S., Brill G., Garcia-Ocana A., Scott D.K. (2021). Nrf2: the master and captain of beta cell fate. Trends Endocrinol. Metab..

[71] Gurlo T., Kim S., Butler A.E., Liu C., Pei L., Rosenberger M. (2019). Pregnancy in human IAPP transgenic mice recapitulates beta cell stress in type 2 diabetes. Diabetologia.

[72] Prentice K.J., Luu L., Allister E.M., Liu Y., Jun L.S., Sloop K.W. (2014). The furan fatty acid metabolite CMPF is elevated in diabetes and induces beta cell dysfunction. Cell Metab..

[73] Liu Y., Prentice K.J., Eversley J.A., Hu C., Batchuluun B., Leavey K. (2016). Rapid elevation in CMPF may act as a tipping point in diabetes development. Cell Rep..

[74] Nolan C.J. (2014). Lipotoxicity, beta cell dysfunction, and gestational diabetes. Cell Metab..

[75] Akirav E.M., Lebastchi J., Galvan E.M., Henegariu O., Akirav M., Ablamunits V. (2011). Detection of beta cell death in diabetes using differentially methylated circulating DNA. Proc. Natl. Acad. Sci. U. S. A..

[76] Kenna L.A., Olsen J.A., Spelios M.G., Radin M.S., Akirav E.M. (2016). beta-Cell death is decreased in women with gestational diabetes mellitus. Diabetol. Metab. Syndr..

[77] Sorenson R.L., Brelje T.C. (2009). Prolactin receptors are critical to the adaptation of islets to pregnancy. Endocrinology.

[78] Le T.N., Elsea S.H., Romero R., Chaiworapongsa T., Francis G.L. (2013). Prolactin receptor gene polymorphisms are associated with gestational diabetes. Genet. Test Mol. Biomarkers.

[79] Newey P.J., Gorvin C.M., Cleland S.J., Willberg C.B., Bridge M., Azharuddin M. (2013). Mutant prolactin receptor and familial hyperprolactinemia. N. Engl. J. Med..

[80] Banerjee R.R., Cyphert H.A., Walker E.M., Chakravarthy H., Peiris H., Gu X. (2016). Gestational diabetes mellitus from inactivation of prolactin receptor and MafB in islet beta-cells. Diabetes.

[81] Pepin M.E., Bickerton H.H., Bethea M., Hunter C.S., Wende A.R., Banerjee R.R. (2019). Prolactin receptor signaling regulates a pregnancy-specific transcriptional program in mouse islets. Endocrinology.

[82] Lucas B.K., Ormandy C.J., Binart N., Bridges R.S., Kelly P.A. (1998). Null mutation of the prolactin receptor gene produces a defect in maternal behavior. Endocrinology.

[83] Ormandy C.J., Camus A., Barra J., Damotte D., Lucas B., Buteau H. (1997). Null mutation of the prolactin receptor gene produces multiple reproductive defects in the mouse. Genes Dev..

[84] Brelje T.C., Parsons J.A., Sorenson R.L. (1994). Regulation of islet beta-cell proliferation by prolactin in rat islets. Diabetes.

[85] Vasavada R.C., Garcia-Ocana A., Zawalich W.S., Sorenson R.L., Dann P., Syed M. (2000). Targeted expression of placental lactogen in the beta cells of transgenic mice results in beta cell proliferation, islet mass augmentation, and hypoglycemia. J. Biol. Chem..

[86] Friedrichsen B.N., Galsgaard E.D., Nielsen J.H., Moldrup A. (2001). Growth hormone- and prolactin-induced proliferation of insulinoma cells, INS-1, depends on activation of STAT5 (signal transducer and activator of transcription 5). Mol. Endocrinol..

[87] Chen H., Kleinberger J.W., Takane K.K., Salim F., Fiaschi-Taesch N., Pappas K. (2015). Augmented Stat5 signaling bypasses multiple impediments to lactogen-mediated proliferation in human beta-cells. Diabetes.

[88] Lee J.Y., Gavrilova O., Davani B., Na R., Robinson G.W., Hennighausen L. (2007). The transcription factors Stat5a/b are not required for islet development but modulate pancreatic beta-cell physiology upon aging. Biochim. Biophys. Acta.

[89] Goyvaerts L., Schraenen A., Lemaire K., Veld P.I., Smolders I., Maroteaux L. (2022). Normal pregnancy-induced islet beta cell proliferation in mouse models that are deficient in serotonin-signaling. Int. J. Mol. Sci..

[90] Kwak S.H., Park B.L., Kim H., German M.S., Go M.J., Jung H.S. (2012). Association of variations in TPH1 and HTR2B with gestational weight gain and measures of obesity. Obesity (Silver Spring).

[91] Demirci C., Ernst S., Alvarez-Perez J.C., Rosa T., Valle S., Shridhar V. (2012). Loss of HGF/c-Met signaling in pancreatic beta-cells leads to incomplete maternal beta-cell adaptation and gestational diabetes mellitus. Diabetes.

[92] Dishi M., Hevner K., Qiu C., Fida N.G., Abetew D.F., Williams M.A. (2015). Early pregnancy maternal hepatocyte growth factor and risk of gestational diabetes. Br. J. Med. Med. Res..

[93] Hamze Z., Vercherat C., Bernigaud-Lacheretz A., Bazzi W., Bonnavion R., Lu J. (2013). Altered MENIN expression disrupts the MAFA differentiation pathway in insulinoma. Endocr. Relat. Cancer.

[94] Jyotsna V.P., Malik E., Birla S., Sharma A. (2015). Novel MEN 1 gene findings in rare sporadic insulinoma--a case control study. BMC Endocr. Disord..

[95] Zhang H., Zhang J., Pope C.F., Crawford L.A., Vasavada R.C., Jagasia S.M. (2010). Gestational diabetes mellitus resulting from impaired beta-cell compensation in the absence of FoxM1, a novel downstream effector of placental lactogen. Diabetes.

[96] Davis D.B., Lavine J.A., Suhonen J.I., Krautkramer K.A., Rabaglia M.E., Sperger J.M. (2010). FoxM1 is up-regulated by obesity and stimulates beta-cell proliferation. Mol. Endocrinol..

[97] Plank J.L., Frist A.Y., LeGrone A.W., Magnuson M.A., Labosky P.A. (2011). Loss of Foxd3 results in decreased beta-cell proliferation and glucose intolerance during pregnancy. Endocrinology.

[98] Qiao L., Saget S., Lu C., Zang T., Dzyuba B., Hay W.W. (2022). The essential role of pancreatic alpha-cells in maternal metabolic adaptation to pregnancy. Diabetes.

[99] Quesada-Candela C., Tuduri E., Marroqui L., Alonso-Magdalena P., Quesada I., Nadal A. (2020). Morphological and functional adaptations of pancreatic alpha-cells during late pregnancy in the mouse. Metabolism.

[100] Szlapinski S.K., Bennett J., Strutt B.J., Hill D.J. (2021). Increased alpha and beta cell mass during mouse pregnancy is not dependent on transdifferentiation. Exp. Biol. Med. (Maywood).

[101] Samols E., Marri G., Marks V. (1965). Promotion of insulin secretion by glucagon. Lancet.

[102] Shiota C., Prasadan K., Guo P., El-Gohary Y., Wiersch J., Xiao X. (2013). alpha-Cells are dispensable in postnatal morphogenesis and maturation of mouse pancreatic islets. Am. J. Physiol. Endocrinol. Metab..

[103] Thorel F., Damond N., Chera S., Wiederkehr A., Thorens B., Meda P. (2011). Normal glucagon signaling and beta-cell function after near-total alpha-cell ablation in adult mice. Diabetes.

[104] Luyckx A.S., Gerard J., Gaspard U., Lefebvre P.J. (1975). Plasma glucagon levels in normal women during pregnancy. Diabetologia.

[105] Scaglia L., Smith F.E., Bonner-Weir S. (1995). Apoptosis contributes to the involution of beta cell mass in the post partum rat pancreas. Endocrinology.

[106] Takahashi M., Miyatsuka T., Suzuki L., Osonoi S., Himuro M., Miura M. (2020). Biphasic changes in beta-cell mass around parturition are accompanied by increased serotonin production. Sci. Rep..

